# *LDJump*: Estimating variable recombination rates from population genetic data

**DOI:** 10.1111/1755-0998.12994

**Published:** 2019-04-04

**Authors:** Philipp Hermann, Angelika Heissl, Irene Tiemann‐Boege, Andreas Futschik

**Affiliations:** ^1^ Department of Applied Statistics Johannes Kepler University Linz Linz Austria; ^2^ Institute of Biophysics Johannes Kepler University Linz Linz Austria

**Keywords:** bioinformatics, change‐point estimation, population recombination rate, regression, R‐package

## Abstract

As recombination plays an important role in evolution, its estimation and the identification of hotspot positions is of considerable interest. We propose a novel approach for estimating population recombination rates based on genotyping or sequence data that involves a sequential multiscale change point estimator. Our method also permits demography to be taken into account. It uses several summary statistics within a regression model fitted on suitable scenarios. Our proposed method is accurate, computationally fast, and provides a parsimonious solution by ensuring a type I error control against too many changes in the recombination rate. An application to human genome data suggests a good congruence between our estimated and experimentally identified hotspots. Our method is implemented in the R‐package *LDJump*, which is freely available at https://github.com/PhHermann/LDJump.

## 1 INTRODUCTION

Recombination is a process during meiosis, which starts with the formation of DNA double‐strand breaks (DSBs) and results in an exchange of genetic material between homologous chromosomes (Baudat, Imai, & de Massy, 2013). The process leads to the formation of new haplotypes and increases the genetic variability in populations. In most species, recombination is concentrated in narrow regions known as hotspots, 1–2 kb in length, flanked by large zones with low recombination or cold regions. Meiotic recombination is a tightly regulated process and is controlled in most mammals by a methyltransferase protein called PR domain zinc finger protein 9 (PRDM9) (reviewed in Baudat et al., 2013; Tiemann‐Boege, Schwarz, Striedner, & Heissl, 2017). PRDM9 binds specific sequence motifs (e.g., the Myers motif) with its zinc finger array and recruits the DSB machinery (SPO11) to the hotspot (reviewed in Tiemann‐Boege et al., 2017). Hotspots vary between species (human vs. chimpanzee [Auton et al., 2012], or mice [Smagulova et al., 2011]), populations within species (human populations like Africans and Europeans [Berg et al., 2010; Pratto et al., 2014; The 1000 Genomes Project Consortium, 2015]), individuals within species (humans [Pratto et al., 2014]), individuals of different sexes (Kong et al., 2010), as well as between viruses (reviewed in Pérez‐Losada, Arenas, Galán, Palero, & González‐Candelas, 2015). Molecular and evolutionary mechanisms of the process of recombination can be better understood with accurate local estimates of the recombination rate (Chan, Jenkins, & Song, 2012; McVean et al., 2004). Moreover, knowledge of the recombination rate variation along DNA sequences improves inference from polymorphism data about e.g., positive selection (Sabeti et al., 2006), or linkage disequilibrium (Hill & Robertson, 1968), and facilitates an efficient design and analysis of disease association studies (McVean et al., 2004). For this purpose, we designed *LDJump*, an algorithm that provides a fast and reliable new estimate of variable genome‐wide population recombination rates by partitioning the DNA sequence into regions with similar recombination. *LDJump* also permits demography to be taken into account.

Methods differing in their genome‐wide coverage and resolution to estimate either active or historical recombination have been developed to estimate recombination rates in humans. Experimental approaches include whole genome sequencing, or SNP typing of pedigrees of at least two to three generations (Coop, Wen, Ober, Pritchard, & Przeworski, 2008; Halldorsson et al., 2016; Kong et al., 2010; Williams et al., 2015), leading to a resolution in the order of tens of kilobases, or less in the more recent studies that included more individuals. Direct measurements in sperm provide high resolution events at the level of a few hundred base pairs, but lack genome‐wide coverage (Arbeithuber, Betancourt, Ebner, & Tiemann‐Boege, 2015; Arnheim, Calabrese, & Tiemann‐Boege, 2007; Kauppi, Jeffreys, & Keeney, 2004). Finally, recombination hotspots have been inferred by the analysis of patterns of linkage disequilibrium (McVean et al., 2004; Myers, Bottolo, Freeman, McVean, & Donnelly, 2005; Myers, Freeman, Auton, Donnelly, & McVean, 2008). The latter approach provides genome‐wide historical recombination events that occurred over thousands of generations in both males and females inferred from polymorphisms characterized in many individuals within a population.

One of the first approaches to infer the population recombination rate *ρ* from patterns of linkage disequilibrium was to compute a lower bound on the number of recombination events (Hudson & Kaplan, 1985; Myers & Griffiths, 2003; Wiuf, 2002). In population genetics, *ρ* is defined as *ρ* = 4*N*_*e*_ *r*, where *N*_*e*_ is the effective population size and *r* the recombination rate per base pair (bp) and generation. Other methods estimate *ρ* via maximum likelihood (Fearnhead & Donnelly, 2001; Kuhner, Yamato, & Felsenstein, 2000) or approximations to the likelihood (Fearnhead & Donnelly, 2002; Hudson, 2001; Li & Stephens, 2003; McVean, Awadalla, & Fearnhead, 2002; Wall, 2004). The former methods rely on simulations using importance sampling (Fearnhead & Donnelly, 2001) or Markov chain Monte Carlo (MCMC) methods (Kuhner et al., 2000) to become computationally feasible. The latter approaches use a composite likelihood (Hudson, 2001), or a modified composite likelihood (McVean et al., 2002). Software implementations such as *LDhat* (Auton & McVean, 2007; McVean et al., 2004) and *LDhelmet* (Chan et al., 2012) are also available. Kamm, Spence, Chan, and Song (2016) extend this approach to account for demographic effects in their software package *LDpop*. Generally, computing approximate likelihoods requires a somewhat smaller computational effort than full likelihoods at the price of a slight loss in accuracy. An improvement of composite likelihood estimators via optimizing the trade‐off between bias and variance has been proposed by Gärtner and Futschik (2016). For a more technical discussion on composite likelihood in general see Varin, Reid, and Firth (2011) and Reid (2013). Other approaches rely on moment estimates or more generally on summary statistics (Batorsky et al., 2011; Hudson, 1987). In Wall (2000, 2004), suitably chosen summary statistics such as the number of haplotypes (haps) are used.

Further well established frameworks to estimate recombination rates include Lamarc (Kuhner, 2006), OmegaMap (Wilson & McVean, 2006), RDP (Martin, Murrell, Golden, Khoosal, & Muhire, 2015), and CodABC (Arenas, Lopes, Beaumont, & Posada, 2015). The latter method (Arenas et al., 2015) applies approximate Bayesian computation (ABC) using 26 summary statistics to estimate constant recombination rates for simulated regions of size up to 300 codons for 100 alignments. With the GUI of RDP (Martin et al., 2015) overall patterns of recombination and testing for hot and cold spots is performed with help from *LDhat* (McVean et al., 2004). Recently, alternative fast estimates of *ρ* that rely on regression on sliding windows have been proposed by Lin, Futschik, and Li (2013) and Gao, Ming, Hu, and Li (2016). Their software implementation is called *FastEPRR* and is recommended by the authors for larger samples consisting of 50 sequences or more.

So far all these previous methods have at least some limitations such as being computationally demanding, not designed for small sample sizes or leading to too many change points in the recombination map. With *LDJump*, we provide a computationally fast and reliable method that provides parsimonious recombination maps. In our approach, we divide the DNA sequence into short segments and estimate the recombination rate per segment via a regression based on the following carefully selected summary statistics: a normalized measure for the number of haplotypes, Watterson's *θ*, normalized measures on pairwise differences, haplotype heterozygosity, neighbour similarity score (NSS; Jakobsen & Easteal, 1996), and the maximal chi‐squared (MaxChi; Smith, 1992). A frequentist segmentation algorithm (Frick, Munk, & Sieling, 2014) is then applied to the estimated rates to obtain change‐points in recombination. The algorithm controls a type I error and provides confidence bands for the estimator. Futschik, Hotz, Munk, and Sieling (2014) use a similar approach to partition DNA sequences into homogeneous segments with respect to GC content. In contrast to Gao et al. (2016), our approach also works well with small sample sizes down to 10 sequences.

Section 2 contains a detailed description of our proposed method. In section 3, we investigate the performance of *LDJump* and compare it with the software packages *LDhat* and *FastEPRR*. Results for the estimation of *ρ* in the presence of demographic effects together with a short comparison to *LDpop* is also provided. Section 2 in the Supporting Information Appendix S1 considers additionally *LDhelmet*, a further well known software package. As a practical illustration, we apply our approach to a well characterized region of the human genome for some human populations. We furthermore estimate population specific recombination maps for the complete human chromosome 16, showing a good overlap between our and experimental estimates of hotspot positions. Finally, we summarize our findings in section 4. Further details on the regression model, bias correction, and more detailed simulation results are provided in Supporting Information Appendix S1.

## 2 MATERIALS AND METHODS

Our approach consists of two steps. First, we fit a regression model from simulated data to estimate constant recombination rates on short segments. Subsequently, we apply a segmentation algorithm to estimate change points in the recombination rate. The algorithm provides a type I error control against over‐estimating the number of identified breakpoints.

### 2.1 Regression model for constant recombination rates

In our model, we used a Box‐Cox (Box & Cox, 1964) transformation *t*(*ρ*) of the population recombination rate *ρ* as our response. This was motivated since the direct use of *ρ* as response would lead to variance heterogeneity. For further details see section 1.2 of the Supporting Information Appendix S1. In order to regress *t*(*ρ*) on summary statistics computed on simulated short DNA segments, we use generalized additive models (GAM) (Wood, 2011) and estimate cubic spline functions *f*_*j*_(*z*_*j*_) for the covariates *z*_*j*_, *j *=* *1, …, *q*. The structure of our GAM is

(1) $$t\left(\rho_{i}\right)=f_{1}\left(z_{i1}\right)+\cdots +f_{q}\left(z_{\mathrm{iq}}\right)$$

for *i *=* *1,…, *n*.

We considered several summary statistics proposed in the literature. We removed those predictors that required a substantial computational burden or led frequently to missing values. Since all remaining summary statistics contributed significantly to the prediction, we chose them as our explanatory variables *z*_*j*_ *, j *=* *1,…, *q*. Table 1 contains all considered summary statistics, providing marks for those selected in our model. Spline functions were used, as modeling the summary statistics as linear and quadratic effects led to less satisfactory results.

**Table 1:** Summary statistics considered for our additive regression model

Variable	Description	Computation
**z**		
* haps*	The number of haplotypes per base pair and per sequence	Haplotype of *pegas* (Paradis, 2010)
* vapw*	Variance of the average pairwise differences per base pair	Convert of *LDhat* (McVean et al., 2004) or self implementation
* apwd*	Average number of pairwise differences per base pair	Convert of *LDhat* (McVean et al., 2004) or self implementation
* wath*	Wattersons's θ per base pair	theta.s of pegas (Paradis, 2010)
* hahe*	Mean of haplotype heterozygosity for each pair of sites	Hs of adegenet (Jombart, 2008)
* MaxChi*	Maximal chi‐squared	PhiPack (Bruen, Philippe, & Bryant, 2006)
* NSS*	Neighbour similarity score	PhiPack of (Bruen et al., 2006)
**n**		
* rsqu*	Mean of *r* ^2^ for each pair of sites	Diseq of genetics (Warnes, Gorjanc, Leisch, & Man,^2013^)
* ldpr*	Mean of D’ for each pair of sites	Diseq of genetics (Warnes et al.,^2013^)
* hats*	Constant recombination rate estimator of a segment	Pairwise of *LDhat* (McVean et al., 2004)
* fgts*	The number of pairs of sites for which the four gametes test indicates a recombination event per base pair	Self implementation
* mean(phi)*	Mean value of the pairwise homoplasy index (PHI) statistic	PhiPack of Bruen et al. (2006)
* var(phi)*	Variance of the PHI‐test statistics	PhiPack of Bruen et al. (2006)
* gcco*	GC content: ratio of guanine and cytosine in the DNA sequence	gc.content of ape (Paradis et al., 2004)

Notes

The section tagged with **z** contains variables that are included in the model using spline functions. The section tagged with **n** contains variables that we did not use due to run time (*rsqu*,* ldpr*,* fgts*), software dependence (*hats*), a high share of missing values compared to other summary statistics (*mean*[*phi*], *var*[*phi*]), or no signicant effect (*gcco*). All selected variables were statistically significant.

For a more detailed description of the regression model, as well as, the selection of explanatory variables see section 1.1 in the Supporting Information Appendix S1.

Since low recombination rates were overestimated on average (and high rates underestimated), we added a bias correction that uses quantile regression of the true vs. the estimated recombination rate on simulated data. For further details on the bias correction, see section 1.3 and Figures S4 and S5 in the Supporting Information Appendix S1.

### 2.2 Segmentation algorithm estimating variable recombination rates

Frick et al. (2014) introduced a method called SMUCE for detecting change points in a function for observations distributed according to an exponential family. This method starts with a constant function and introduces successively additional jumps, as long as they lead to a significant increase in the likelihood. Using likelihood ratio tests, the probability of overestimating the number of change‐points is controlled subject to a user specified type I error probability *α*. For a given number of jumps, the best fitting locally constant function is chosen by maximizing the likelihood. We use this method with local estimates $\hat{\rho }$ as input. For a general overview on multiple change‐point detection see Niu, Hao, and Zhang (2016).

In the first step *LDJump* divides the DNA sequence into *k* short segments. Summary statistics are computed separately for each segment and inserted into our regression model to estimate a local transformed recombination rate. The back‐transformed rates follow an approximate normal distribution (natural scale of *ρ*, see Supporting Information Appendix S1, section 1.2) and are used as input for the change point estimator. In our simulations, the use of the back‐transformed rates led to a better detection of hotspots compared to the transformed rates.

## 3 RESULTS

We used the software package scrm of Staab, Zhu, Metzler, and Lunter (2014) to simulate samples of populations with variable recombination rates and converted its output to *fasta*‐files with the software package ms2dna of Haubold and Pfaffelhuber (2013). In this section we compare *LDJump* with *LDhat*,* LDhelmet*,* FastEPRR*, and *LDpop*. We consider both constant and variable recombination rates and look at the performance and the run time. The run time comparison is based on one core of an Intel Xeon E5‐2630v3 2.4 1866, with 64 GB DDR4‐2133 RAM. Our analysis was performed in R (R Development Core Team, 2018). Note that all mentioned software packages can also be applied on several cores in parallel.

### 3.1 Constant recombination rate estimation

We first focus on a constant recombination rate on a DNA segment. In our simulations, *LDJump* is compared with the functions pairwise of *LDhat* and max_lk of *LDhelmet* following the default guidelines. The chosen sample sizes (number of sequences) were (10, 16, 20), and the sequence lengths (1,000, 2,000, 3,000) base pairs. For each of these nine setups, we simulated under 111 different values of *ρ* ∈ [0,0.1] yielding a total of 999 simulated scenarios. The population mutation rate was chosen as *θ* = 0.01.

**Figure 1:**
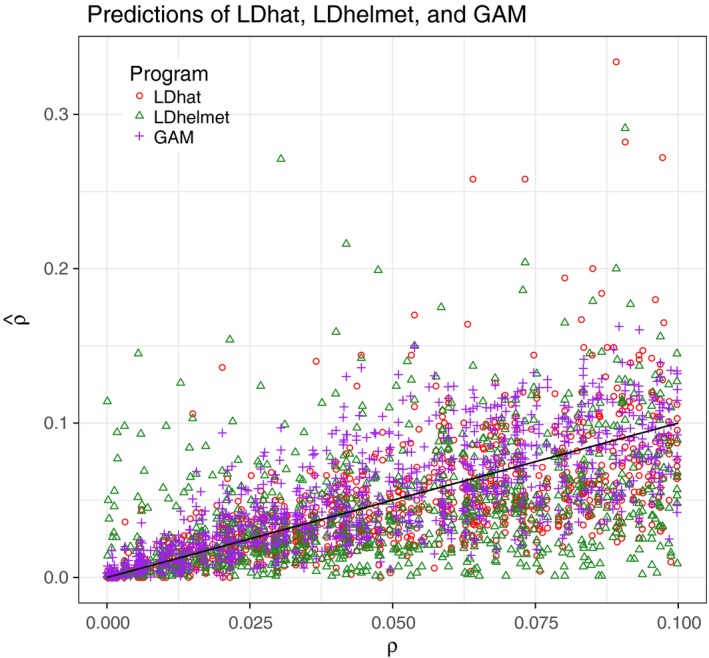
Constant recombination rates: true recombination rates vs. predicted values from *LDhat* (red dots), *LDhelmet* (green triangles), and *LDJump* (purple plus signs). The black line indicates a perfect prediction [Colour figure can be viewed at wileyonlinelibrary.com]

Using the root mean squared error ($\text{RMSE}=\sqrt{\frac{1}{n}\sum_{i=1}^{n}{\left(\hat{\rho_{i}}-\rho_{i}\right)}^{2}}$) and the coefficient of determination *R* ^2^, we compare the accuracy of the mentioned methods. We visualize the estimators and the true values in Figure 1 along with a diagonal black line indicating a perfect fit. Both prediction measures show a slightly better fit of the generalized additive model (purple plus signs: higher *R* ^2^ of 0.5661; smaller RMSE of 0.0241) compared with the software packages *LDhat* (red dots: 0.4447; 0.0290) and *LDhelmet* (green triangles: 0.2095; 0.0360).

### 3.2 Variable recombination rate estimation

For humans, large fractions of recombination events are concentrated on short segments which are called hotspots (reviewed in Arnheim et al., 2007; Tiemann‐Boege et al., 2017). Following the literature, we define recombination hotspots as genomic regions that exceed the background rate by more than a threshold factor of five for a length of up to 2 kb (McVean et al., 2004).

We investigate how well hotspots are detected by our method and simulated two types of setup for variable recombination rate estimation: simple setups (sequences of length 10 and 20 kb with one hotspot) and natural setups (sequences of length 1 Mb containing 15 hotspots) both using a mutation rate *θ* of 0.01. These scenarios were investigated with different background rates, sample sizes, hotspot intensities, and hotspot lengths. When comparing our approach with *LDhat* (using the function rhomap) and *LDhelmet*, we followed recommendations for both programs and used 10^6^ iterations for the reversible‐jump MCMC procedure, sampled every 4,000 iterations, chose a burn‐in of 10^5^, and different block penalties of 0, 5, and 50. For the computations with *LDhelmet*, we also used a window size of 50 SNPs, and 11 Padè coefficients. The results for *FastEPRR* were obtained using *winLength = stepLength* (segment lengths) of 500, 1,000, 1,500, and 2,000 base pairs. In our analysis, we applied the function smuceR available in the R‐package stepR (Hotz & Sieling, 2016) to estimate the change‐points for our method. We took *α* = 0.05 as error probability but also considered *α* = 0.1 and 0.01 to see how sensitive the results are with respect to the specified *α*.

#### 3.2.1 Simple setups

We simulated samples of sizes (10, 16, 20) with sequence lengths of 10 kb and 20 kb. Our 15 considered background recombination rates were chosen equidistantly within 0.001, 0.03. We considered hotspot intensities of 5‐, 10‐, 15‐, 20‐, 40‐fold the background recombination rate. The length of the hotspots varied among $\left\{\frac{1}{5},\frac{1}{10},\frac{1}{20},\frac{1}{35},\frac{1}{50}\right\}$‐times the sequence length. Due to the large number of resulting setups and the computation times of *LDhelmet* and *LDhat*, we restricted this analysis to two replicates per sample yielding in total 4,500 simulated recombination maps. We approximated the RMSE (root mean squared error) as our quality measure, and computed the estimation errors on an equidistant grid of 1,000 positions along the sequences.

Table S2 in Supporting Information Appendix S1 summarizes the performance of the aforementioned methods *LDhat* (Auton & McVean, 2007), *LDhelmet* (Chan et al., 2012), *FastEPRR* (Gao et al., 2016), the first published version of *LDhat(v1)* (McVean et al., 2004), and *LDJump*. As shown in the Supporting Information Appendix S1 (section 1.4), segment lengths of at least 400 bp are needed for a good performance of *LDJump*. Following this recommendation, our method performs equivalently or slightly better than *LDhat*, and outperforms also *LDhelmet*. The choice of *α* did not have a large effect under the considered scenarios. Similarly, the block penalty does not affect considerably the performance of *LDhat*. With *LDhelmet* on the other hand, the choice of the block penalty strongly influences the performance. Compared to *LDhelmet*, the performance of *LDJump* and *LDhat* turned out to be more constant across simulations. Indeed, the standard deviation of the RMSE is more than 50% lower with *LDJump* than that of *LDhelmet*. With *FastEPRR*, approximately 57%, 5%, 4%, and 2% of the computations terminated due to errors using segment lengths of 500, 1,000, 1,500, 2,000, respectively. When *FastEPRR* provided estimates, the performance was comparable with *LDJump*. A more detailed graphical display of the performance of *FastEPRR* with respect to segment lengths can be found in Figure S9 in section 2 of the Supporting Information Appendix S1.

**Figure 2:**
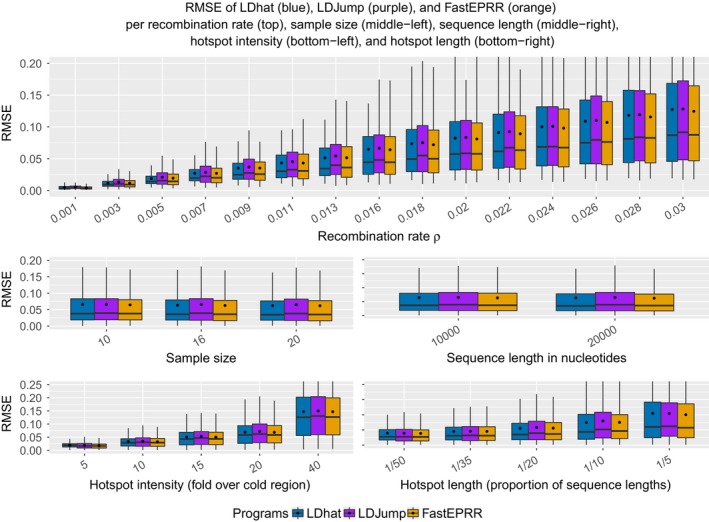
Comparison of the methods (*LDhat* (blue), *LDJump* (purple), and *FastEPRR* (orange) for different true recombination rates (top), sample sizes (middle‐left), sequence lengths (middle‐right), hotspot intensities (bottom‐left), and hotspot lengths (bottom‐right). Mean values are shown as black dots [Colour figure can be viewed at wileyonlinelibrary.com]

Figure 2 contains separate results for different sample sizes, recombination rates, hotspot intensities and lengths, as well as, sequence lengths. We applied *LDJump* with 10 segments and a type I error probability of 5%. Hence, the considered segments had a length of 1,000 and 2,000 (for 10 kb and 20 kb, respectively) nucleotides. We used *FastEPRR* with a window length of 2 kb in order to achieve a small number (32) of runs terminating due to errors. We obtained similar values for the RMSE with *LDJump*,* LDhat*, and *FastEPRR* for all considered hotspot intensities, and sequence lengths. *LDhelmet* reaches a similar level of accuracy only for samples of size 20, hotspot lengths of 1/5 and high background recombination rates (not shown).

#### 3.2.2 Natural setups

We simulated samples with 16 sequences and sequence lengths of 1 Mb. The setups varied in the background rate which was chosen among 13 equidistant values between 0.001 and 0.01. The 15 hotspots were evenly distributed along the sequence and had different intensities between 8‐ to 40‐fold the background rate. Every setup was replicated 20 times. The same mutation rate *θ* = 0.01 was chosen for all setups. In our simulations, we focused on the methods that performed best for the simple scenarios. When using *FastEPRR* the segment lengths of 1 kb were terminated without producing estimates for 88% of our simulated complex data sets. For this reason, we focused on comparing *LDJump* and *LDhat*. With *LDhat* we used a block penalty of 50 which led to smallest RMSE under the simple setups. Additional information on the performance of *FastEPRR* based on the nonterminating runs can be found in section 3 of the Supporting Information Appendix S1. However, it should be noted that a high proportion of missing outputs may lead to a biased quality assessment, especially if the missing probability depends on features of the data set that affect the performance of the estimate.

### 3.3 Quality assessment

We took the weighted RMSE as measure of quality. It is defined as

$$\text{WRMSE}=\sqrt{\sum_{i=1}^{n}w_{i}{\left({\hat{\rho }}_{i}-\rho_{i}\right)}^{2}},$$

with *w*_*i*_ denoting the length of the estimated segment *i* divided by the total sequence length. Furthermore, we considered the proportion of correctly identified hotspots (PCH), also known as positive predictive value. A hotspot is counted as “correctly identified” if it has a nonempty intersection with a detected hotspot (i.e., a region with at least 5‐fold background recombination rate). The proportion of correctly identified background rates (PCB) has been defined analogously and is often named negative predictive value. Finally, we compare the average performance in terms of the mean of the latter two performance measures by AP = (PCH + PCB)/2.

To identify the best choices for the bias correction and segment lengths, we applied *LDJump* with *k* = 500, 1,000, 1,500, and 2,000 segments and estimated the recombination maps using the 0.25, 0.35, 0.45, and 0.5 quantiles in the bias correction (see Supporting Information Appendix S1 section 1.3). Notice that segment lengths resulting from the chosen values of *k* are 2 kb, 1 kb, 666 and 500 bp. As hotspot lengths are either 1 or 2 kb, the scenario with *k* = 1,500 is most challenging as the hotspot boundaries will systematically differ from the segment boundaries. A direct comparison with *LDhat* using a block penalty of 50 (based on the results from the simple setups) is provided.

**Figure 3:**
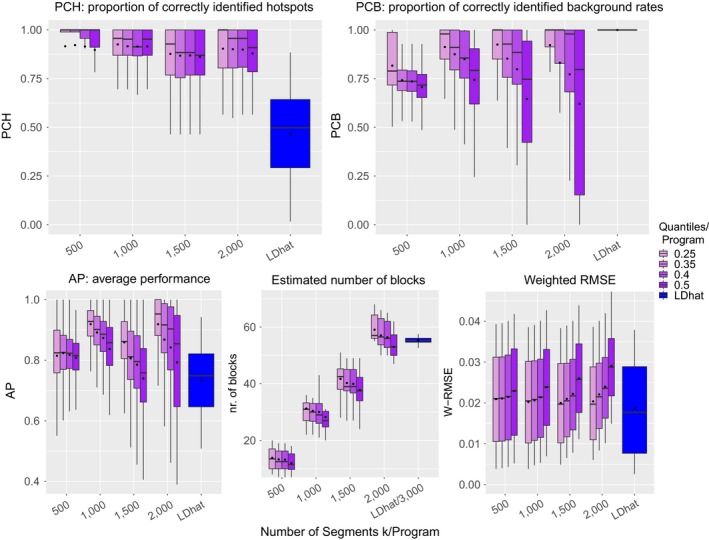
Natural setups: quality assessment is performed for *LDhat* and *LDJump* based on the proportion of correctly identified hotspots (PCH, top‐left), the proportion of correctly identified background rates (PCB, top‐right), the average performance (AP = (PCH+PCB)/2, bottom‐left), the estimated number of blocks (bottom‐middle), and the weighted RMSE (bottom‐right). Based on 13 setups with 20 replicates these measures are computed for *LDJump* using different numbers of initial segments *k* (500, 1,000, 1,500, 2,000) and compared with the results from rhomap of *LDhat* using a block penalty of 50 [Colour figure can be viewed at wileyonlinelibrary.com]

The different choices of *k* are displayed by the first four groups of boxplots in Figure 3. For each of these four groups, quantiles of 0.25, 0.35, 0.4, and 0.5 are used in the bias correction and are presented in different colours. The rightmost bar in each panel (in blue) summarizes the result of *LDhat*. From top‐left to bottom‐right, we show PCH, PCB, AP, the estimated number of blocks, and the weighted RMSE. We can see that our method has very high hotspot detection rates irrespective of *k* with even less variability in performance than *LDhat*. On the other hand, *LDhat* has very high PCB proportions. In comparison, the best PCB values for *LDJump* are obtained for the smallest quantile.

As an overall measure, we display the mean of PCH and PCB as AP in the bottom‐left panel. It turns out that AP is larger for *LDJump* regardless of the tuning parameters. In the bottom‐middle panel we can see that the number of estimated blocks of *LDJump* depends on *k*. When using 500 segments, the estimated number of blocks is below 31, which is the true number of blocks in the recombination map (of 15 hotspots). For 1,000 segments the estimated number of blocks is very similar to the true number of blocks, but as *k* gets larger the number of blocks is slightly overestimated. *LDhat* estimated many more blocks using the block penalty with smallest RMSE under simple setups (50). In fact, the number of change points in recombination tended to be larger by a factor of more than 3,000. Although a choice between zero and fifty is recommended in the software manual, we guess that the number of change points with *LDhat* could be decreased by increasing the block penalty.

The bottom‐right plot shows the weighted RMSE as an overall quality measure showing a similar level of accuracy across *k* and compared with *LDhat*. A more detailed investigation reveals that our method estimates hotspot rates more precisely, but provides less accurate estimators of the background recombination rate.

Our results also show that our method is fairly robust with respect to tuning choices. This is also true for *k* = 1,500, where the hotspots have an unfavourable location compared to the design segment boundaries. To obtain a reasonable tradeoff between sensitivity (PCH) and specificity (PCB), we chose segment lengths of 1 kb (based on 1,000 segments of sequence length 1 Mb) and a quantile of 0.35 in the bias correction, which seemed to a good choice with *LDJump*. We obtained an error proportion of more than 88% using *FastEPRR* for the natural setups. We provide a comparison of the error‐free results in Figure S11 in Supporting Information Appendix S1 section 4. Based on this smaller number of results for *FastEPRR*,* LDJump* performs favourable in terms of the WRMSE and PCH, but has lower PCB compared to *FastEPRR*.

### 3.4 Populations under different levels of genetic diversity

**Figure 4:**
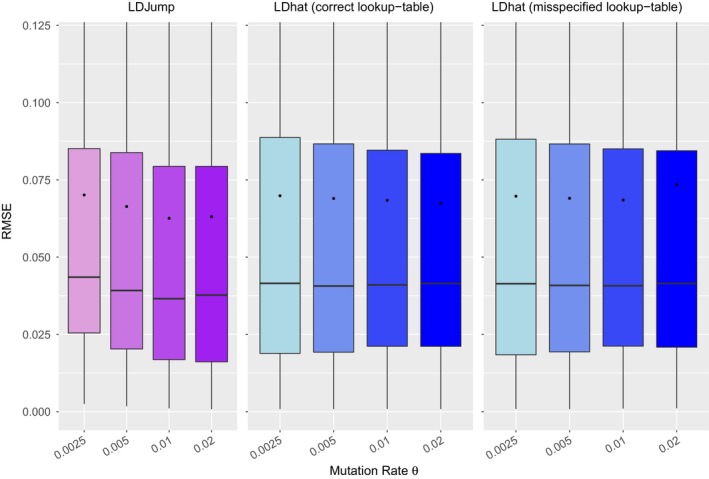
Simple setups: accuracy of estimates for different levels of genetic diversity introduced by different mutation rates *θ*. *LDJump* (first panel) is compared with *LDhat*. Misspecified values of *θ* are also considered: Indeed, *LDJump* was trained only under the mutation rate *θ* = 0.01. For *LDhat*, we compare the performance under different mutation rates (second panel) and under misspecification assuming that the true mutation rate is equal to 0.01 (third panel, misspecified for *θ* ≠ 0.01) [Colour figure can be viewed at wileyonlinelibrary.com]

Since natural populations differ in the level of genetic diversity, we simulated samples under different mutation rates *θ* ∈ {0.0025, 0.005, 0.01, 0.02}. For each mutation rate we simulate the same setup as for the comparisons under simple setups, see section 3.2. In Figure 4 we compare the performance based on the RMSE of *LDJump* (first panel) with *LDhat*. For both methods, the influence of a misspecified *θ* has also been investigated. We used *LDJump* with segment lengths of 1 kb, and the regression model calibrated under the mutation rate *θ* = 0.01. Thus the model is misspecified when the true *θ* ≠ 0.01. For *LDhat*, results obtained using the true value of *θ* are displayed in the second panel, and results under misspecification in the third panel of Figure 4.

The estimation accuracy of *LDJump* improves with increasing mutation rates (or higher genetic diversity) due to the higher information available per segment. Interestingly, *LDhat* benefits less from increased levels of genetic diversity. A misspecified *θ* had little effect on the performance of *LDhat*.

Based on these simulations we also evaluate the influence of the SNP density on the performance of *LDJump*. Figure S8 of the Supporting Information Appendix S1 provides box plots illustrating the performance in terms of the RMSE depending on the estimated mean number of SNPs per base pair within a simulated segment. Our results suggest that a higher SNP density results in more accurate estimates. If a segment contains only one or zero SNPs, then our software implementation imputes estimates based on the neighbouring segments.

### 3.5 Populations under demography

It has been observed by McVean et al. (2002), Chan et al. (2012) and Smith (2005) that ignoring population demography by wrongly assuming a constant population size leads to biased estimates of recombination. As a remedy, Kamm et al. (2016) computed two locus likelihoods under a known variable population size. *LDJump* permits the natural inclusion of any type of demography or even range of demographic scenarios by simply fitting our regression model under suitable scenarios.

We illustrate this approach and consider a scenario that involves a bottleneck followed by a rapid population growth. This scenario has also been used by Kamm et al. (2016). More precisely, we chose time‐dependent population sizes as follows:

(2) $$\eta \left(t\right)=\left\{\begin{array}{cc} 100, & -0.5<t\leq 0\\ 0.1, & -0.58<t\leq -0.5\\ 1, & t\leq -0.58 \end{array}\right.$$

Time is scaled in coalescent units and the simulations were again performed with scrm (Staab et al., 2014). Johnston and Cutler (2012) analyzed a similar demographic scenario and showed in their paper (we did not replicate these results) that *LDhat* infers spurious recombination hotspots when falsely assuming a constant population size.

With *LDJump*, we fitted our regression model using samples simulated under the demographic model (2). We used the same explanatory variables as under neutrality, but added Tajima's *D* (Tajima, 1989) as an additional explanatory factor to the regression model. This additional variable had significant effect on the model fit in our ANOVA, suggesting that choosing summary statistics dependent on demography can help to improve the accuracy of our estimates. We did not change the parameters in the Box‐Cox transformation compared to the constant population size model.

**Figure 5:**
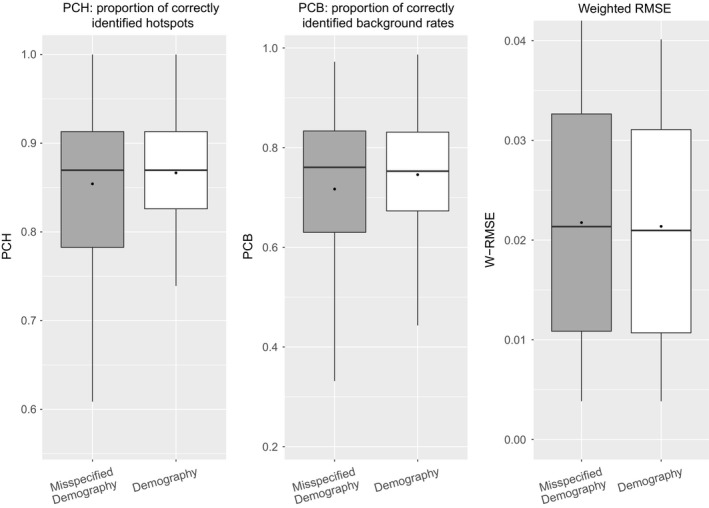
Performance of *LDJump* under the demographic model (2) (grey boxes) compared with the results under *misspecified demography* (white boxes), where a neutral model was incorrectly assumed. We set the segment lengths to 1 kb for these comparisons and use the quantile of 0.35 in the bias correction. We provide box plots for the quality measures PCH (left), PCB (centre), and WMRSE (right)

To see what can be gained by explicitly considering an underlying demography, we simulated samples under the demographic model (2). For these samples, we estimated recombination maps using the regression models trained either under neutrality (misspecified “old” model) or under demography (correctly specified “new” model). More specifically *LDJump* has been applied with segment lengths of 1 kb and a quantile of 0.35. The accuracy of these models was then compared in terms of the indicators PCH, PCB, and WRMSE. The results are shown in Figure 5. When using the correct demographic model, the hotspot detection rate and the proportion of correctly identified regions with background recombination rate increase and show less variability. We also found the WRMSE to be equal or slightly smaller when using the correct demographic model again with less variability.

#### 3.5.1 Comparison with *LDpop*

This section contains a comparison with the recently introduced software package *LDpop* (Kamm et al., 2016). This package enables demographic effects to be considered when computing lookup tables which can then be used within *LDhat* or *LDhelmet*. Hence, we calculated lookup tables for 16 sequences under the demography model (2). We evaluated *LDJump* and *LDpop* based on simulated samples under the demography model (2) with populations of sample size 16 and sequence length of 30 kb. This setup contains two hotspots, both of length 1 kb with intensities 20‐ and 30‐fold higher than the background recombination rates. This setup was simulated 10 times under the same 13 different background rates as in the natural setup, see section 3.2.2. In Table 2 we compare the mean, median, and standard deviation per background recombination rate between the two methods. We can see that under very small background rates *LDpop*, has a lower average RMSE; however, for background rates of at least 0.0039 we obtained smaller mean and median RMSE with *LDJump*.

**Table 2:** Demographic scenario: Mean, median, and *SD* of the RMSE are listed for *LDJump* (with segment lengths of 1 kb, type I error of 0.05 and the 0.35 quantile) and *LDpop* using samples simulated under demography

ρ	Program	Mean	Median	*SD*
0.0010	*LDJump*	0.0080	0.0074	0.0014
	*LDpop*	0.0066	0.0067	0.0005
0.0013	*LDJump*	0.0108	0.0096	0.0032
	*LDpop*	0.0090	0.0091	0.0005
0.0022	*LDJump*	0.0170	0.0160	0.0027
	*LDpop*	0.0154	0.0154	0.0006
0.0027	*LDJump*	0.0194	0.0190	0.0014
	*LDpop*	0.0190	0.0190	0.0006
0.0039	*LDJump*	0.0273	0.0272	0.0004
	*LDpop*	0.0275	0.0277	0.0009
0.0045	*LDJump*	0.0316	0.0314	0.0005
	*LDpop*	0.0318	0.0320	0.0008
0.0054	*LDJump*	0.0383	0.0378	0.0013
	*LDpop*	0.0388	0.0390	0.0008
0.0062	*LDJump*	0.0439	0.0434	0.0012
	*LDpop*	0.0441	0.0441	0.0005
0.0071	*LDJump*	0.0495	0.0494	0.0007
	*LDpop*	0.0512	0.0512	0.0003
0.0080	*LDJump*	0.0561	0.0560	0.0007
	*LDpop*	0.0579	0.0583	0.0008
0.0085	*LDJump*	0.0604	0.0600	0.0017
	*LDpop*	0.0614	0.0614	0.0007
0.0091	*LDJump*	0.0645	0.0637	0.0023
	*LDpop*	0.0653	0.0654	0.0006
0.0100	*LDJump*	0.0699	0.0694	0.0012
	*LDpop*	0.0723	0.0720	0.0008

Notes

The results are obtained based on 10 replicates of different background rates. 16 sequences of length 30 kb containing two hotspots both of length 1 kb with intensities of 20 and 35 are simulated.

### 3.6 Run time

Obtaining estimates of recombination can be computationally demanding, especially for a larger number of sequences, and separate analyses for several populations. Hence, we also provide a comparison with respect to run time (in seconds) between *LDhat(v1)*,* LDhat*,* LDhelmet*,* FastEPRR*, and *LDJump*. We first considered simple setups using our simulated sequences of length 20 kb. Again, we looked at different block penalty choices and at different numbers of atomic segments *k* for *LDJump* in Supporting Information Appendix S1: Table S3. We computed as summaries the mean (top), median (middle), and SD (bottom) of our measured run times. We can see that especially *LDhat* and *LDhelmet* run 10 to 50 times longer than *FastEPRR* and even up to 100 times longer than *LDJump*. In terms of speed, the *LDhat(v1)* is only slightly slower; however, *LDhat (v1)* estimates are considerably less accurate (see Supporting Information Appendix S1: Table S1) *LDJump* also turns out to be faster than *FastEPRR* for all considered number of segments *k*.

In Table 3 we show the mean, median, and SD of run times in seconds based on natural setups. On average *LDJump* turns out to be between 340 and 1,400 times faster than *LDhat*. Choosing larger values of *k* increases the run time for *LDJump*. The increase of the run time is approximately linear with the number of segments chosen. In contrast to our approach, the run times strongly depend on the underlying recombination rates with *LDhat*, leading to a considerable difference between the median and mean of times. In Supporting Information Appendix S1 (section 4), we compare the run times for various background rates and different values of *k*. The computations for estimating recombination under demography took 70 times longer (without considering the computation time of the lookup table) using *LDpop* (on average 1,357 s) compared to *LDJump* (on average 19 s). Overall, *LDJump* provides a particularly attractive combination of performance and run time.

**Table 3:** Mean ($\overset{¯}{x}$) median (*x*_0.5_), and SD of the run times (in seconds) for *LDhat2* and *LDJump* under our natural setups

	*LDhat2*	*LDJump k*			
		500	1,000	1,500	2,000
$\overset{¯}{x}$	77,237	55	111	168	226
*x*_0.5_	122,396	55	111	168	225
SD	2,434	3	3	5	11

Note

For *LDJump* we provide values depending on the number of predefined segments *k*.

### 3.7 Validation of *LDJump* computed hotspots with active recombination hotspots

We first tested our algorithm on a 103 kb region on human chromosome 21. Therefore, we sampled the region between SNPs rs10622653 and rs2299784, a region in the human genome in which recombination was characterized at high resolution by sperm typing (Tiemann‐Boege, Calabrese, Cochran, Sokol, & Arnheim, 2006). Taking data from The 1000 Genomes Project Consortium (2015), we randomly chose 25 individuals for each of five subpopulations from five European regions (TSI, FIN, IBS, GBR, CEU). We reformatted files from vcf‐ to fasta‐format with the R packages (Knaus & Grünwald, 2017; Paradis, Claude, & Strimmer, 2004) using two sequences per (diploid) sample and the reference sequence 80.37 (GRCH37) from The 1000 Genomes Project Consortium (2015). We applied *LDJump* with a segment length of 1 kb, chose the 35% quantile for the bias‐correction, and used the demography model. When we ignored demography and applied *LDJump* under a neutral scenario, we obtained a higher amount of false positive candidates (see Figure S12 in the Supporting Information Appendix S1). Our considered demography model (2) is rather simple, and we stress that *LDJump* can also be applied under any demographic scenario by training the regression model with a suitable setup.

**Figure 6:**
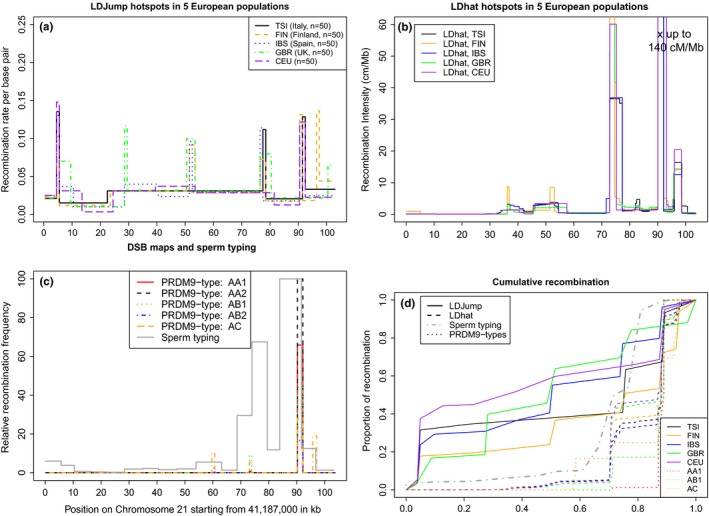
(a) Estimated recombination map of five different European populations (Italy, Finland, Spain, United Kingdom, Northern Europeans from Utah‐CEU) on chromosome 21:41187000–41290679 (GRCH37). (b) The estimated *LDhat* maps (Auton & McVean, 2007) for the same populations are retrieved from the 1000G (http://www.internationalgenome.org/data-portal/search?q=recombination). (c) Relative recombination based on measured double strand break (DSB) intensities for five different individuals representing active recombination from Pratto et al. (2014). Moreover, we plot in grey (solid line) the estimated crossover frequency of the same 103 kb region on chromosome 21 based on sperm typing 13 intervals ∼5 kb in size, taken from Tiemann‐Boege et al. (2006). The *y*‐axis was scaled to the maximum of the DSB intensity or crossover frequency within that region, respectively. (d) Accumulation of recombination with increasing sequence for each method and population estimated with *LDJump* (solid lines), *LDhat* maps from the 1000G (dashed lines), DSB intensities for three individuals (dotted lines) (Pratto et al., 2014) and sperm‐typing (dash‐dotted line)(Tiemann‐Boege et al., 2006). The colour coding remains the same for the five European populations [Colour figure can be viewed at wileyonlinelibrary.com]

We observed that in the region from 70–90 kb within the investigated 103 kb, the *LDJump* recombination maps across populations overlap to the map obtained experimentally using sperm typing in Tiemann‐Boege et al. (2006) (see panel a of Figure 6) and with the double strand break (DSB) map (see panel b of Figure 6, Pratto et al., 2014). Note that the latter two maps represent active male recombination hotspots); whereas, the LD‐based estimated maps using *LDJump* and *LDhat* capture historical recombination averaged between males and females. However, in region 50 to 60 kb we observe only historical hotspots detected by *LDJump* and *LDhat*, see panel b of Figure 6. We do not observe these hotspots in active recombination measures of sperm typing (Tiemann‐Boege et al., 2006) or DSB (Pratto et al., 2014). Moreover, we also find hotspots unique to *LDJump*, which are either found in all considered subpopulations (e.g., at 5 kb for all five populations) or in specific populations (e.g., at 30 kb only for GBR). Additionally, we estimated the cumulative recombination frequency in the region (accumulation of recombination with increasing sequence for each method and population) in panel d of Figure 6. For most of these recombination measures the majority of the recombination (>65%) takes place in 25% of the sequence.

We further tested the performance of *LDJump* within a larger genomic region to validate our method. For this purpose, we applied *LDJump* to the entire chromosome 16, and consider separate samples of 50 sequences from five populations (GBR, TSI, IBS, FIN, CEU) taken from The 1000 Genomes Project Consortium (2015). For the data preparation we used the software package *vcftools* (Danecek et al., 2011) and then ran a parallel version of *LDJump* with segment lengths of 1 kb for each population recombination map. We obtained these results in about 16 hr using in total 15 cores of an Intel Xeon E5‐2630v3 2.4 1866, with 64 GB DDR4‐2133 RAM.

**Figure 7:**
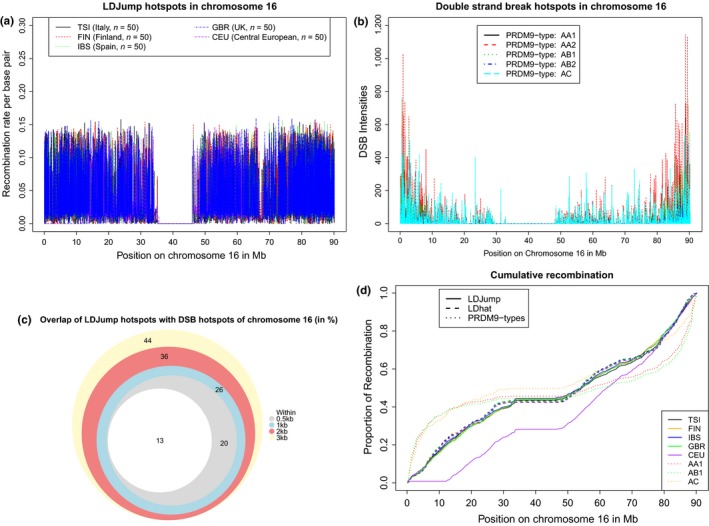
(a) Estimated recombination map for chromosome 16 of four European populations with 50 randomly sampled sequences of the 1000 Genomes Project using *LDJump* under the demography model and segment lengths of 1 kb. The results of the Italian sample are plotted in black, the Finnish sample in dashed red, the Spanish population in dotted green and the British one in dash‐dotted blue. (b) Double‐strand break maps taken from Pratto et al. (2014) of chromosome 16 for five individuals with different PRDM9‐types. Here, the different colours and line types represent different individuals (AA1, solid‐black; AA2, dashed‐red; AB1, dotted‐green; AB2, dash‐dotted‐blue; AC, long‐dashed‐cyan). (c) Overlap between detected DSB hotspots and the hotspots identified by *LDJump*. With *LDJump*, we define hotspots as regions with more than five times the estimated background rate. The DSB hotspots were taken from Pratto et al. (2014). We looked at overlaps between *LDJump* and the DSB hotspots that occurred with at least one European population (white areas). To assess the level of accuracy, we added segments of length 0.5 (grey), 1 (cyan), 2 (red), and 3 (yellow) kb left and right to the DSB region boundaries. The comparison is performed for all PRDM9‐types considered in Pratto et al. (2014). The total number of DSB hotspots of all PRDM9‐types is 2889 of which 866 were not within 3 kb of a *LDJump* hotspot. (d) Accumulation of recombination within chromosome 16 with increasing sequence for each method and population estimated with *LDJump* (solid lines), *LDha*t maps from the 1000G (dashed lines), DSB intensities for three individuals (dotted lines) (Pratto et al., 2014). The colour coding remains the same for the five European populations. The shift in cumulative recombination of the CEU compared to the other populations results from a lack of information up to position 10 MB [Colour figure can be viewed at wileyonlinelibrary.com]

In panel a of Figure 7 we show the estimated recombination maps under the demography model (2) for chromosome 16 with the Italian population (TSI) in black, the Finnish sample in dashed red (FIN), the Spanish sample (IBS) in dotted green, the British population (GBR) in dash‐dotted blue, and the Central European population (CEU) in long‐dashed purple. Overall, we observe population specific hotspots, but also hotspots present in more than one population as is also observed in genome‐wide DSB maps (Figure 7, panel b) (Pratto et al., 2014).

Furthermore, we evaluated the agreement of the estimated recombination hotspot locations using *LDJump* with the DSB map hotspots. For identifying *LDJump* hotspots we use a simple heuristic to define the average background rate. More specifically, we chose the mean of all estimates $\hat{\rho }$ that fall below the median. This should give a downward biased estimate. With *LDJump*, we again defined regions as hotspots as those with a 5‐fold higher estimated background rate. The DSB hotspots were selected by making use of the indicator variables provided by (Pratto et al., 2014). Given that DSB‐hotspots are very narrow, yet the resolution of DSB into a crossover can occur with 3–5 kb, we added segments of different length (0, 0.5, 1, 2, 3 kb) left and right to the DSB‐hotspot regions and calculated the respective number of detected hotspots per PRDM9‐type. The total number of DSB hotspots for AA1, AA2, AB1, AB2, and AC is 2889 (Pratto et al., 2014). We counted a hotspot as jointly detected, if an overlap between DSB‐hotspot and a *LDJump* hotspot occurred in at least one of the five populations (FIN, IBS, GBR, TSI, CEU). We display the number of jointly detected hotspots (augmented by segments of different lengths) via a Venn diagram in panel c of Figure 7. Notice that the number of hotspots estimated by *LDJump* for all considered populations is in total 8,237, and therefore approximately 3‐fold higher than the number of DSB‐hotspots. Our analysis shows that on average about 44% of the DSB hotspots (when adding 3 kb segments to these regions) overlapped with at least one of the estimated *LDJump* population hotspots. These proportions are in accordance with the comparison of LD‐based recombination maps and DSB‐hotspots (Pratto et al., 2014) with an overlap of 56%. For chromosome 16, we calculate on average about 49% overlap between *LDhat* and DSB‐hotspots (adding 3 kb segments).

## 4 DISCUSSION

We introduced a new method called *LDJump* to estimate heterogeneous recombination rates along chromosomes from population genetic data. Our approach splits a given DNA sequence into segments of proper length in a first step. Subsequently, we use a generalized additive regression model to estimate the constant recombination rates per segment. Then, we apply a simultaneous multiscale change‐point estimator (SMUCE) to estimate the breakpoints in the recombination rates across the sequence. We provide detailed comparisons of our method with the recent reversible jump MCMC methods *LDhat* and *LDhelmet*, as well as, the regression‐based method *FastEPRR*. Our estimates are very fast, perform favourably in the detection of hotspots, and show similar accuracy levels as the best available competitor for simple and natural setups, respectively. These comparisons show that *LDJump* is a powerful tool to explore recombination rates in organisms with narrow recombination hotspots; for example, PRDM9 defined hotspots in most mammals.

We validated our method by computing hotspots in several human populations and compared the estimated hotspots with recombination intensities measured by sperm‐typing and double‐strand break maps. Within the region of 70–100 kb *LDJump* computed hotspots that mainly agree with hotspots detected at high resolution with sperm typing and Chip immuno‐precipitation (DSB map), as well as, with *LDhat* maps.

*LDJump* also revealed population specific hotspots not present in the active recombination maps (~30 and 50 kb), but partially present in historical maps inferred by *LDhat*. Given the lack of active recombination at position ~50 kb (absence of this hotspot in sperm typing and in the DSB maps for the 2 European donors carrying the PRDM9 allele A, as well as the donor with African descent [carrying the PRDM9 allele C]), we hypothesize that this estimated hotspot might represent a historical hotspot that became extinct. Alternatively, it could be a population‐specific hotspot given that its intensity varies among different European populations. In order to test this latter hypothesis, active recombination maps from different populations are required.

Not all population specific hotspots inferred by *LDJump* overlap with population specific *LDhat* hotspots. The reason could be related to the different sample sizes used (*LDJump* included only a subset of individuals) or the difficulties of *LDhat* screening small sample sizes. The latter might explain the presence of hotspots at position 100 kb but absent in *LDJump* (except FIN) and DSB maps (except PRDM9‐type AC). Finally, we also observed a region with little congruence at position ~10 kb not detected by *LDhat*.

Differences between hotspot rates estimated from LD patterns compared to estimates based on sperm typing have also been observed by Jeffreys and Neumann (2009). This might be caused by the short life‐span of hotspots and their rapid evolution in intensity and genomic position among populations and species (Coop & Myers, 2007; Jeffreys, Cotton, Neumann, & Lam, 2013; Myers et al., 2010). In fact, only ~56% of historical hotspots determined by LD agree with genome‐wide DSB maps (Pratto et al., 2014). Our large‐scale validation on chromosome 16 shows that about 44% of the DSB‐hotspots (in total 2,889) were also found by LDJump (in total 8,237) using five European populations.

Fine‐scale population specific differences with respect to recombination events have also been highlighted in studies by Kong et al. (2010), Berg et al. (2011), Fledel‐Alon et al. (2011), and Pratto et al. (2014). Given all this, our observed differences are likely due to underlying biological features.

We have implemented our approach as an R‐package called *LDJump*, which can be freely downloaded from https://github.com/PhHermann/LDJump. In our simulations, we obtained particularly good results when applying our method with segment lengths of 1 kb and a bias correction using the default quantile of 0.35.

In conclusion, *LDJump* is a fast algorithm which is able to detect narrow hotspots at high accuracy using segments of approximately 1 kb length. Moreover, we also show that *LDJump* can be applied on populations under demography. We validated our method on a 103 kb region of human chromosome 21, as well as, the whole chromosome 16 and found a good congruence by comparing *LDJump* hotspots with recombination hotspots measured with sperm typing or Chip immuno‐precipitation (DSB map).

## AUTHOR CONTRIBUTION

P.H. and A.F. designed the model and implemented the model into the R package. P.H. and A.F. focused on the statistical aspects and I.T.B. and A.H. on the biological aspects. All authors wrote and commented on the manuscript.

## Data Availability

*LDJump* has been implemented as an R package which can be downloaded and installed from Github (https://github.com/PhHermann/LDJump). We also provide example files and a manual in this repository. We downloaded the data of chromosome 16 from ftp://ftp.7011000genomes.ebi.ac.uk/vol1/ftp/release/20130502 and uploaded an R‐script with details on the data management, as well as the hotspot locations for the estimated population recombination maps to Github (https://github.com/PhHermann/Hermann_et_al_2018_LDJump). We downloaded the data for the application on chromosome 21 from http://phase3browser.7051000genomes.org/Homo_sapiens/Location/Overview?r=21:41187000-41290679 using 50 sequences of the five European populations IBS, GBR, TSI, FIN, and CEU. We provide details on the regression model, bias correction, choice of segment lengths, detailed quality assessments and run time comparisons in the Supporting Information Appendix S1 to the online publication.
