# Supplementary material for: LDJump: Estimating variable recombination rates from population genetic data
Source: Mol Ecol Resour. 2019 Apr 4;19(3):623–38. doi: 10.1111/1755-0998.12994 (PMC6519033; doi:10.1111/1755-0998.12994)
Supplement: Supplementary file 1 [file MEN-19-623-s001.pdf]

Supporting Information for online Publication on LDJump: Estimating Variable Recombination Rates from Population Genetic Data

Philipp Hermann[1], Angelika Heissl[2], Irene Tiemann-Boege[2], and Andreas Futschik*[1]

[1]Department of Applied Statistics, Johannes Kepler University Linz, Austria.
[2]Institute of Biophysics, Johannes Kepler University Linz, Austria.

1 Regression Model

Data management for computing the summary statistics was conducted with the R-packages `Biostrings,adegenet,ape` [Pages et al., 2016, Jombart, 2008, Paradis et al., 2004].
We simulated populations with {10, 16, 20} individuals and sequence lengths of {500, 1000, 2000, 3000, 5000} bp. The recombination rates per base pair were simulated from uniform distributions on the intervals {[0,0.01], [0.01,0.02], [0.02,0.05], [0.05,0.1], [0.1,0.2]} and used for every combination of population size and sequence length. For the first setup we simulated 100 recombination rates in [0,0.01]. Subsequently, a population with 10 individuals and a sequence length of 500 nucleotides was simulated using each of these 100 values. This procedure was conducted with all setups that involved different population sizes and sequence lengths. Hence, the simulated data consists 8000 samples, where 1 sample was removed due to the lack of a recombination and mutation event. The mutation rate θ was set 0.01 per base pair in these simulations as well as the computations with *LDhat* and *LDhelmet*. In natural populations GC-biased gene conversion is associated with recombination [Birdsell, 2002] and modifies the GC content in regions with active recombination (reviewed in [Duret and Galtier, 2009]). To the best of our knowledge we are not aware of simulators being capable of reproducing this process. Therefore, we do not use GC content related summaries in our regression model.

1.1 Coefficients & Effect Plots

Figure 1 contains graphical representations of the influence of the summary statistics on the recombination rate. The plots from top-left to bottom-right represent the estimated cubic

*Corresponding Author: andreas.futschik@jku.at

spline functions for the variables *haps, vapw, apwd, hahe, wath, MaxChi*, and *NSS*. The 95% confidence interval of the effect is plotted with dashed lines.

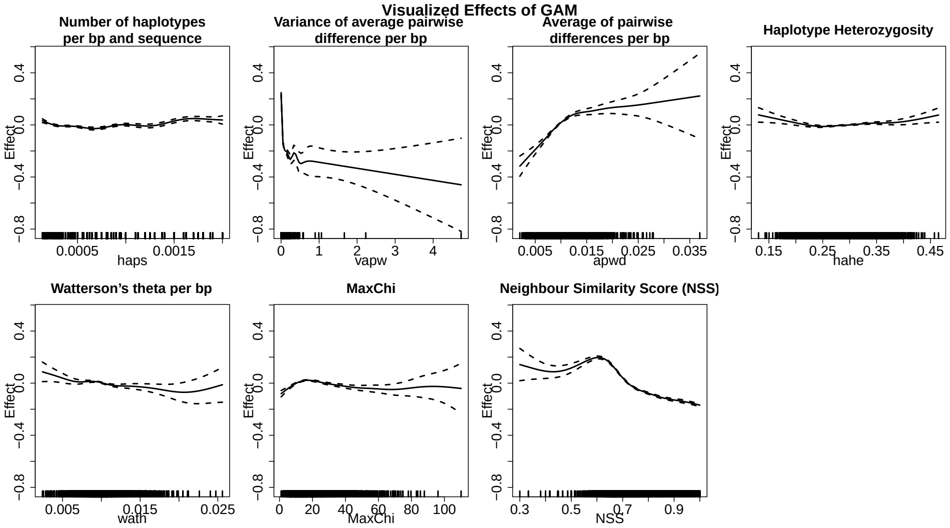

Figure 1: Univariate spline estimates (effects) of the variables in our generalized additive regression model are shown.

Table 1 contains the estimated parameters of the regression model for the summary statistics with significant effects (represented with asterisks). The first two columns show the coefficients and the standard deviation of the quadratic functions and columns three and four the *EDF* and *ref.df* of the cubic spline functions. The quality of fit measure R^2 (0.76) shows a high model fit based on the simulated data.

1.2 On the model assumptions of variance homogeneity and normality

We use the Box-Cox transformation [Box and Cox, 1964] (1) given as

$$t(\rho, \gamma, \epsilon) = \begin{cases} \frac{(\rho+\epsilon)^\gamma - 1}{\gamma} & \text{for } \gamma \neq 0 \\ \ln(\rho + \epsilon) & \text{for } \gamma = 0. \end{cases} \tag{1}$$

This transformation performed best under our considered transformations including also logarithmic and exponential transformations. In order to tune the model with respect to homogeneity and normality of the residuals as well as high prediction accuracy, we compared the performance under different combinations of parameters. The considered grid of values for γ and ϵ for the Box-Cox transformation (1) was $\{0.001, 0.01, 0.05, 0.1, 0.25, 0.5, 0.75, 1\}$ and $\{0, 0.01, 0.02, 0.03, 0.04, 0.05, 0.06, 0.07, 0.08, 0.09, 0.10, 0.15\}$, respectively.
To assess the quality of fit, the plots of Figure 2 are produced with the chosen model and the trained data. The left plot of Figure 2 shows the scatter plot of the predicted values (y-axis) and

Variable	EDF	(Ref.df)
(Intercept)	-1.58	$(0.00)^{***}$
s(haps)	8.25	$(9.00)^{***}$
s(vapw)	8.95	$(9.00)^{***}$
s(apwd)	5.94	$(9.00)^{***}$
s(hahe)	5.13	$(9.00)^{***}$
s(wath)	6.94	$(9.00)^{***}$
s(MaxChi)	6.98	$(9.00)^{***}$
s(NSS)	8.13	$(9.00)^{***}$
	R^2	0.76
	Num. obs.	7427

$^{***}p < 0.001$, $^{**}p < 0.01$, $^{*}p < 0.05$

Table 1: Coefficients of summary statistics estimated via a generalized additive model to explain the recombination rate. EDF refers to the estimated degrees of freedom and Ref.df are the degrees of freedom used in the tests reported.

the true values (x-axis) of the simulated recombination rates (both transformed). By dividing the grid of recombination rates into 15 segments we can compute the standard deviations for the predictions in this interval. The ratio between the standard deviation and the mean of the standard deviations of all intervals is visualized in the middle plot of Figure 2 together with an estimated smoothing spline. The standard deviations differed up to 15% from the mean, the largest deviation being in [0.0148, 0.0259] (down) and [0.1429, 0.1571] (up). Further computations with 10 to 25 segments show robustness with respect to the number of segments, and maximum deviation to the mean of 20%. The right plot of Figure 2 shows the QQ-plot for the residuals of the model.

Figure 3 contains heat maps, illustrating slight deviations from the model assumptions of the GAM models. Each panel has its own color key and is calibrated, with green boxes indicating a good performance in terms of the criterion. The x-axis of each plot contains the values of ϵ and the y-axis the values of γ. The top-left and the top-right panel show the sum of squared and the sum of absolute differences of the standard deviation to their means, respectively. Small values are coded in dark green and indicate a small deviation from variance homogeneity. Greater sums of squared/absolute differences are visualized with brighter colors. Values of γ in a range of 0.35 - 0.5 with $\epsilon = 0$ and γ in a range of 0.1 - 0.5 with $\epsilon = 0.1$ are seen as possible candidates for a proper transformation.

Normality of the residuals is considered in the bottom-left panel. Here, Shapiro-Wilk statistics are calculated with values close to 1 coded in green color. The standard implementation of this test in [R Development Core Team, 2017] is restricted to 5000 observations. Therefore, we drew 100 different samples of 5000 residuals and computed the mean of these 100 Shapiro-Wilk statistics. We can observe a similar pattern as for the variance homogeneity comparisons except for the combinations with $\epsilon = 0.1$. The quality of the regression model in terms of R^2 also

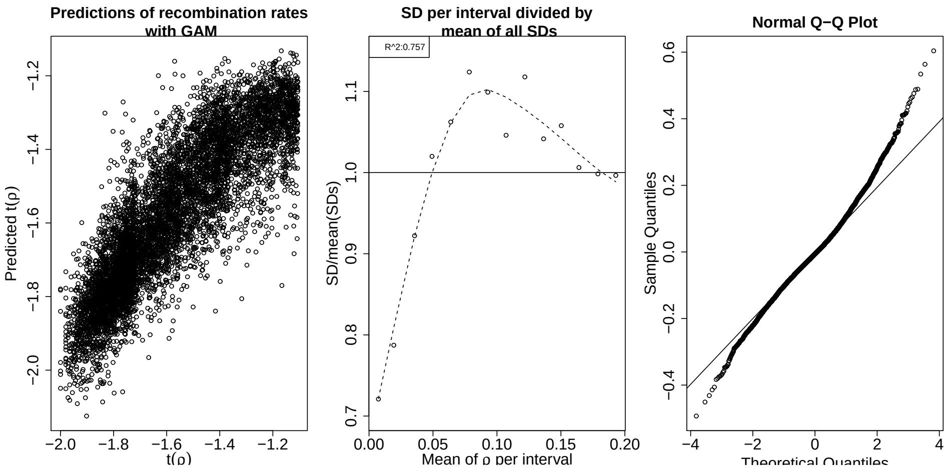

Figure 2: Plot of predicted versus true values (left), graphical tests for variance homogeneity (middle) and normality of residuals (right) of the chosen GAM model.

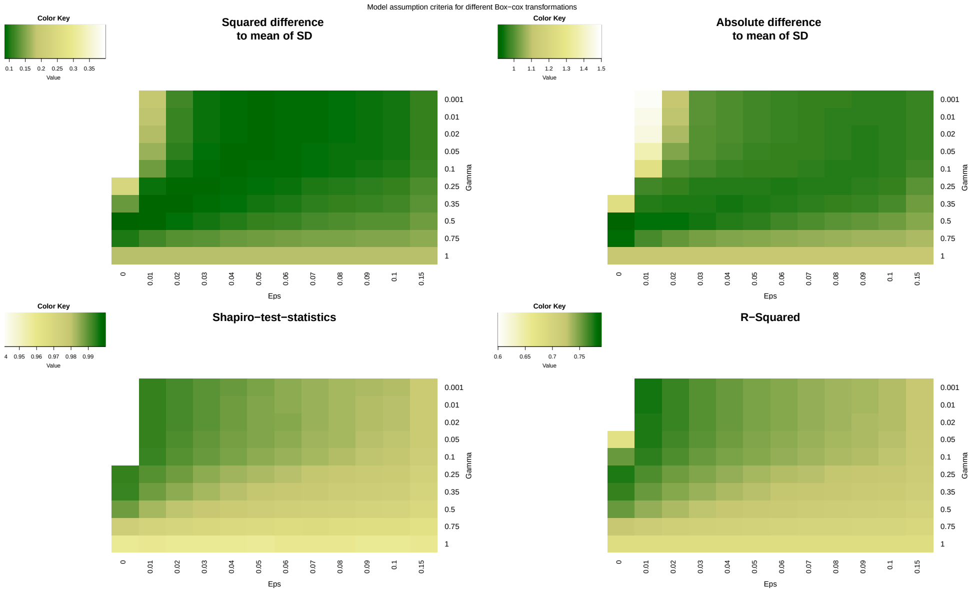

Figure 3: Comparison of model assumptions of Box-Cox transformed ρ under different values for γ and ϵ.

points to the same choice of the parameters γ and ϵ. We finally chose $\gamma = 0.5$ and $\epsilon = 0$ due to the much better performance in terms of the variance homogeneity measures given slightly smaller value of R^2 and very similar value for the Shapiro-Wilk statistics.

1.3 Bias Correction and Homoscedasticity Check

We applied a simulation based bias correction due to an observable bias especially for setups with small background rates. Therefore, we simulated recombination maps of length 1000 kb (1Mb) with in total 15 hotspots of lengths of 1kB (7) and 2kb (8). These recombination maps differ in 10 equidistant background rates between 0.001 and 0.011 with 15 replicates. The hotspots are between five and forty folds of the background recombination rates.
By estimating ρ with k = 1000 we use the systematic overlap of hotspot boundaries and segment boundaries to compare the estimator with the true value. This comparison (transformed scale) is provided in left plot of Figure 4 with a solid black diagonal line as perfect fit. Note that due to the overrepresentation of small recombination rates we have sampled as many background rates as hotspot rates in the recombination map. This yields approximately 4600 observations. We sampled the background rates uniformly from all background rates. Visual inspections reveal an overestimation of the background rate as well as an underestimation of very high ρ. A correction of these patterns is performed with quantile regressions where the estimated recombination rates explain the true recombination rates. The result of the estimated quantile regression for the 0.25 (orange), 0.35 (blue), 0.4 (green), and 0.5 (red) quantile, respectively, is given in Figure 4. On the right hand side of Figure 4 the residuals of the quantile regression models are plotted starting with the 0.25 quantile (top) and ending with 0.50 quantile (bottom). Values smaller than -2 after bias-correction are set to -2, such that they equal to zero after the back-transformation.

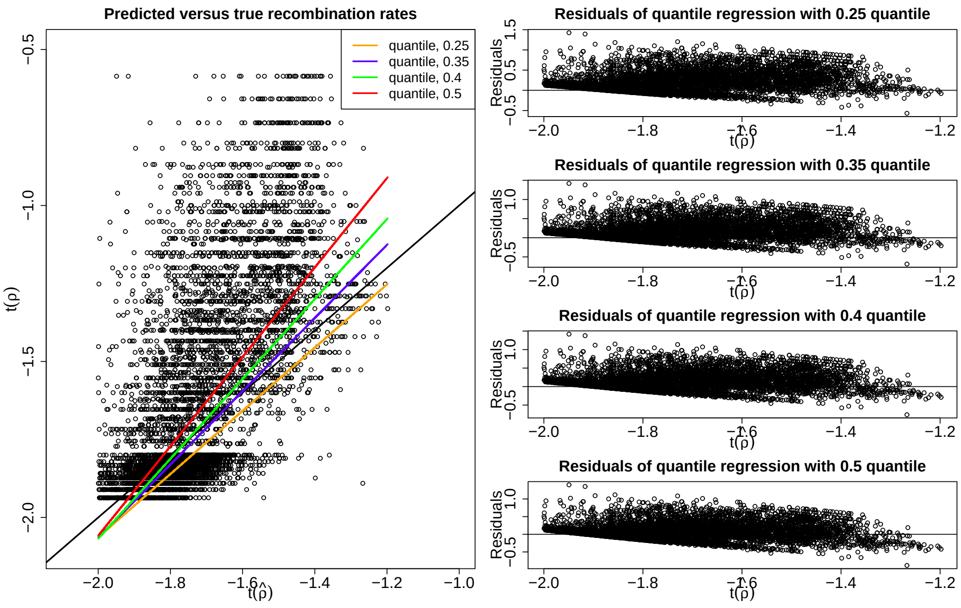

Figure 4: Left: Estimated versus true recombination rates based on recombination maps from simulations containing 15 hotspots of lengths 1 and 2kb. Predictions based on quantile regressions with 0.25 (orange), 0.35 (blue), 0.4 (green), and 0.5 (red) are added in this plot. Right: Residuals originating from the three quantile regressions provided for diagnostic purposes.

Figure 5 provides a comparison between *LDJump* with (grey) and without (purple) bias correction, and *LDhat2* (blue). Three samples with different background recombination rates of 0.001 (left), 0.0054 (middle), and 0.01 (right) are presented in dotted black lines. Segment lengths were chosen to be 1kb with the quantile chosen 0.35 in the bias correction (see supporting information section 1.3) and a type-I error probability of 0.05. The bias-correction decreases the bias in the background rates and increases the intensities of the estimated hotspots.

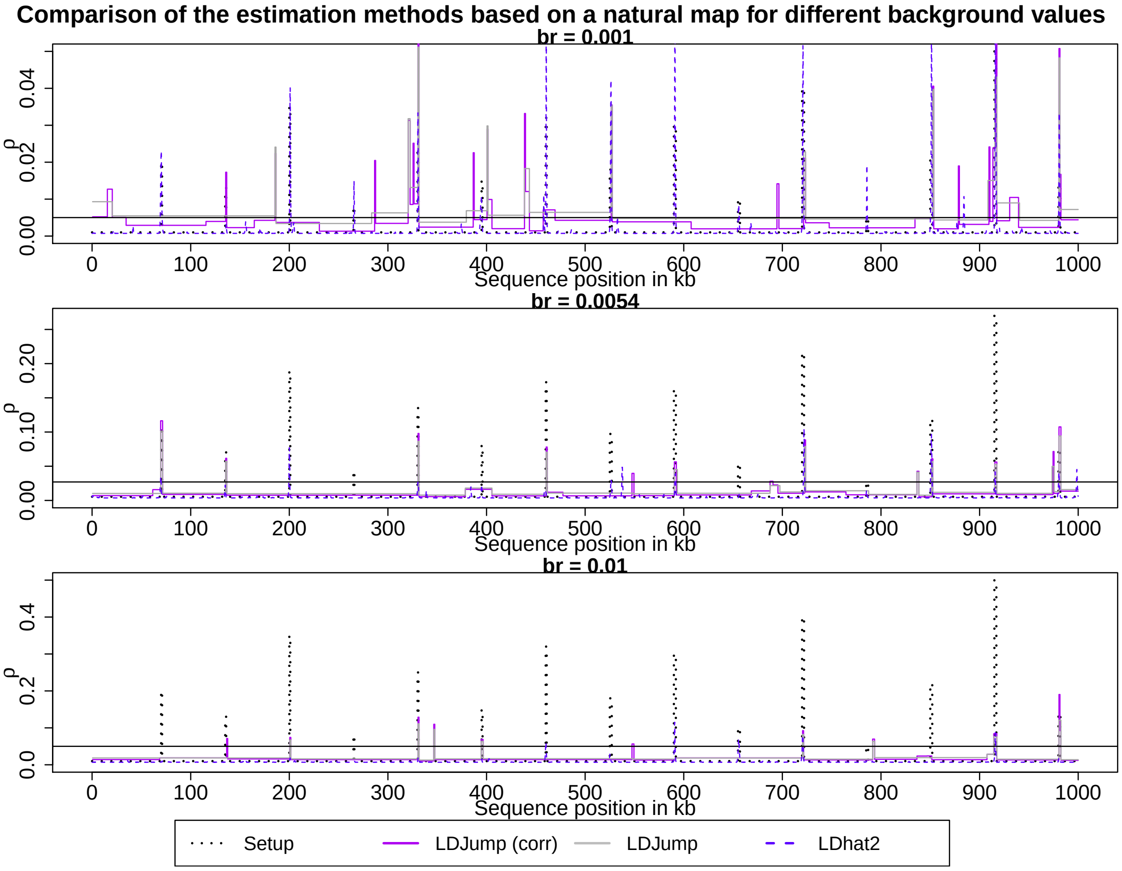

Figure 5: Estimated recombination maps using *LDhat* and *LDJump*, both with and without our bias-correction. The chosen setups differ in the background rates (0.001 (topp), 0.0054 (middle), and 0.01 (bottom)). The true recombination map (black dotted lines) contains 15 hotspots. Horizontal lines represent the hotspot threshold (5·background rate).

The SMUCE estimator requires homoscedastic observations [Frick et al., 2014]. Similar to the approach in supporting information section 1.2, we analyze the homogeneity of the recombination rates by comparing the variance of the recombination rates in different intervals. Here, we divide the range of [0,0.2] in 32 equidistant segments. For each segment we compute the variance of the corrected (and back-transformed) recombination rates. By dividing the variance of each segment with the mean of all variances, we have a measure of the variability of the variances along the considered recombination rates. In Figure 6 we show ratios of variances divided by the mean of variances for all 20 considered intervals with an estimated smoothing

spline for the four quantiles, 25% (left), 35% (middle-left), 40% (middle-right), and 50% (right). The difference of the variance to the mean variance only exceeds 20 percentage points in terms of variances for the first quartile in 3 intervals and for two intervals of the median. When comparing the standard deviations (dashed lines) we can see that these deviations are less than (or slightly above) 15 percentage points (in absolute values) for (almost) all considered quantiles in the correction.

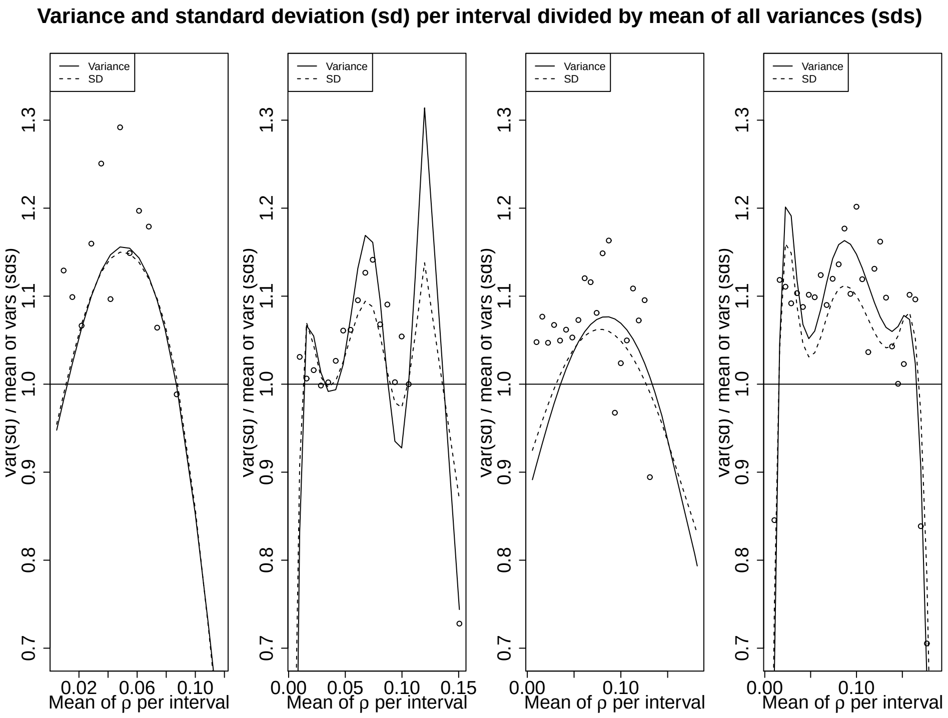

Figure 6: Graphical test for the homogeneity of the estimated recombination rates per quantile used in the quantile regression of the bias-correction (left: 0.25, left-middle. 0.35, right-middle: 0.4, right: 0.5). Variances are computed for 32 intervals of recombination rates between 0 and 0.2. The ratio of the variances divided by the overall mean of variances is plotted. The same approach is applied and visualized in terms of the standard deviation (dashed lines).

1.4 Segment Lengths using *LDJump*

An important tuning parameter of *LDJump* is the number of segments k on which our summary statistics are computed. We chose k between 10 and 50 (yielding segment lengths between 200 and 2000 base pairs depending on the overall sequence length). Figure 7 shows the RMSE depending on the segment length for three different sample sizes. It suggests to choose segments of at least 400 bp. This observation is consistent across the considered sample sizes. The figure also suggests that larger samples only improve the performance under very small segment lengths up to 400 bp. As noted above, we do not recommend to apply *LDJump* under such

small segment lengths. Our considered type-I error probabilities (0.01, 0.05, and 0.1) did not affect these results.

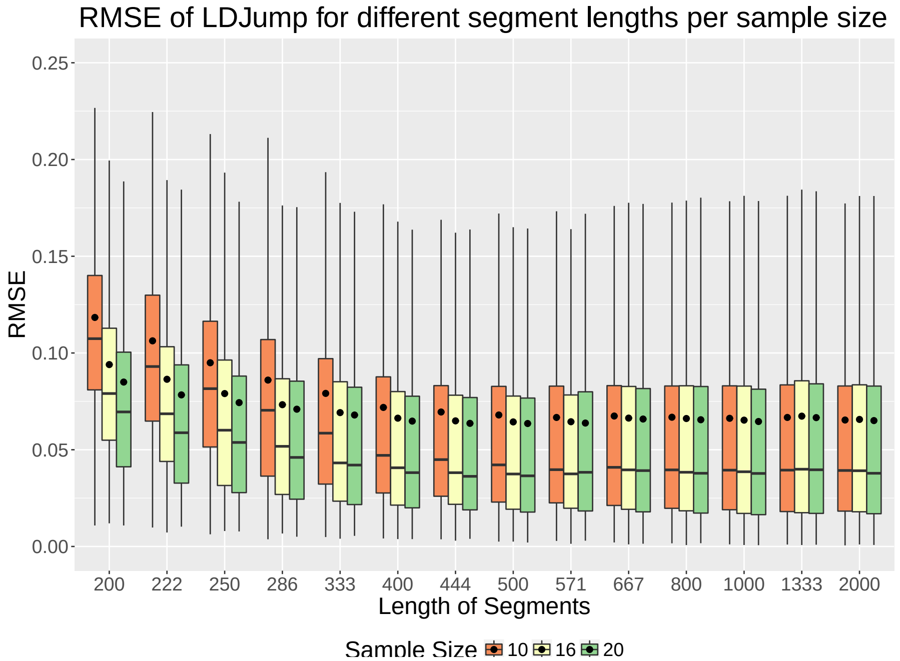

Figure 7: Comparison of the quality of fit of *LDJump* for different segment lengths, distinguished between the considered sample sizes. For the sake of comparability we refrain from plotting the results below segment lengths of 286 bp.

1.5 *LDJump* assessed under different Levels of Genetic Diversity

Figure 8 contains a quality assessment of *LDJump* under different SNP densities. Based on simulated samples under different mutation rates we compute intervals with different SNP densities per base pair and plot these intervals. Hence, we can conclude that the quality of *LDJump* increases with on average higher SNP densities, i.e. more information present per segment.

2 Detailed Quality Assessment for Simple Setups

In Table 2 we provide a detailed quality assessment between the considered methods for simple setups. More specifically, we computed the mean, median, and standard deviation (across simulations) of the RMSE for *LDhat(v1)* (column 3), *LDhat* (c. 4), *LDhelmet* (c. 5), *FastEPRR* (c. 6-9, with different segment lengths) and *LDJump* (c. 10-15 with different numbers of user-defined segments k). The results using different block penalties for *LDhat, LDhelmet* along with different type I error probabilities for *LDJump* are listed in separate rows.

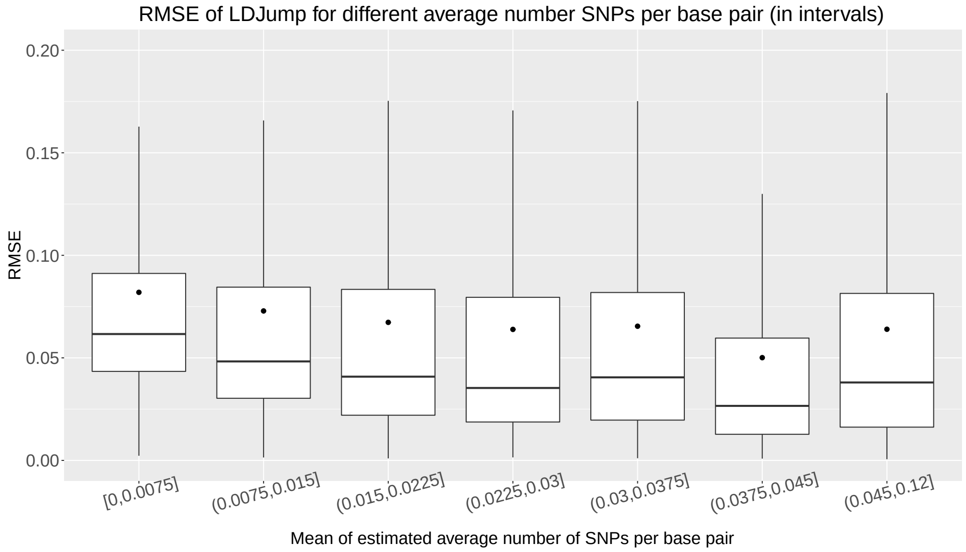

Figure 8: RMSE of *LDJump* using simulated populations under *simple* setups with different mutation rates $\theta = \{0.0025, 0.005, 0.01, 0.02\}$. We compare the performance of *LDJump* depending on the mean number of SNPs per bp.

We compare the performance of *FastEPRR* under the simple setups with respect to segment lengths in Figure 9. Here, we can see the increasing variation based on the estimation results of larger segments. In contrast, the median per group decreases with segment length.

3 Detailed Quality Assessment for Natural Setups

Figure 10 shows our considered quality measures depending on the background recombination rates. We provide the average performance over 20 replicates. We can see that *LDhat* has constant PCB and decreasing PCH as the background rate increases. *LDJump* shows constant values for PCH and slightly increasing PCB for higher background rates. The overall measure AP slightly increases for *LDJump* and decreases for *LDhat* with increasing background rates, respectively. The weighted RMSE is also plotted. It can be seen that *LDhat* leads to a slightly smaller weighted RMSE with decreasing differences for larger ρ.

	bpen	*LDhat(v1)*	*LDhat*	*LDhel*	*FastEPRR* (Segment Length) 500	1000	1500	2000	α	*LDJump* (Number of Segments) 10	15	20	25	30
	0	0.158	0.064	0.286					0.1	0.066	0.067	0.066	0.066	0.066
$\bar{x}$	5	0.132	0.064	0.234	0.053	0.057	0.061	0.063	0.05	0.065	0.067	0.065	0.066	0.066
	50	0.078	0.064	0.094					0.01	0.065	0.067	0.065	0.065	0.066
	0	0.138	0.036	0.247					0.1	0.039	0.040	0.038	0.039	0.040
$x_{0.5}$	5	0.100	0.036	0.169	0.039	0.034	0.035	0.036	0.05	0.039	0.040	0.038	0.039	0.041
	50	0.049	0.036	0.044					0.01	0.039	0.040	0.038	0.039	0.041
	0	0.115	0.076	0.227					0.1	0.076	0.078	0.077	0.076	0.075
SD	5	0.121	0.076	0.224	0.053	0.066	0.072	0.074	0.05	0.076	0.078	0.077	0.075	0.074
	50	0.102	0.076	0.145					0.01	0.075	0.077	0.076	0.075	0.074

Table 2: Mean ($\bar{x}$), median ($x_{0.5}$) and SD of the RMSE for *LDhat(v1)*, *LDhat*, *LDhelmet* (LDhel), *FastEPRR*, and *LDJump* under *simple* setups. Different block penalties (*bpen*) have been tried for *LDhat(v1), LDhat, LDhelmet*. Different segment lengths have been applied with *FastEPRR*, and different number of segments as well as type I error probabilities α considered for *LDJump*.

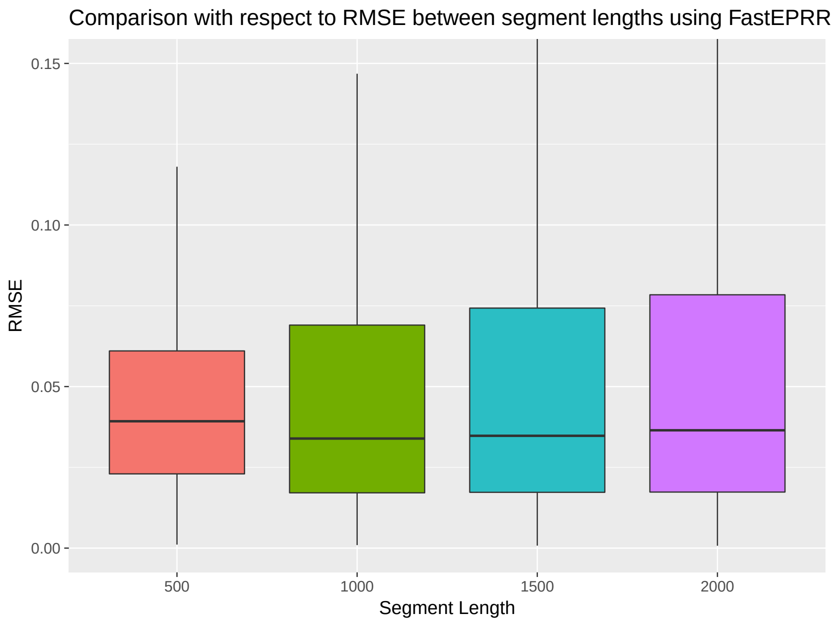

Figure 9: Comparing the RMSE of `FastEPRR` based on different segment lengths of 500 (red), 1000 (green), 1500 (blue), and 2000 (purple) under simple setups.

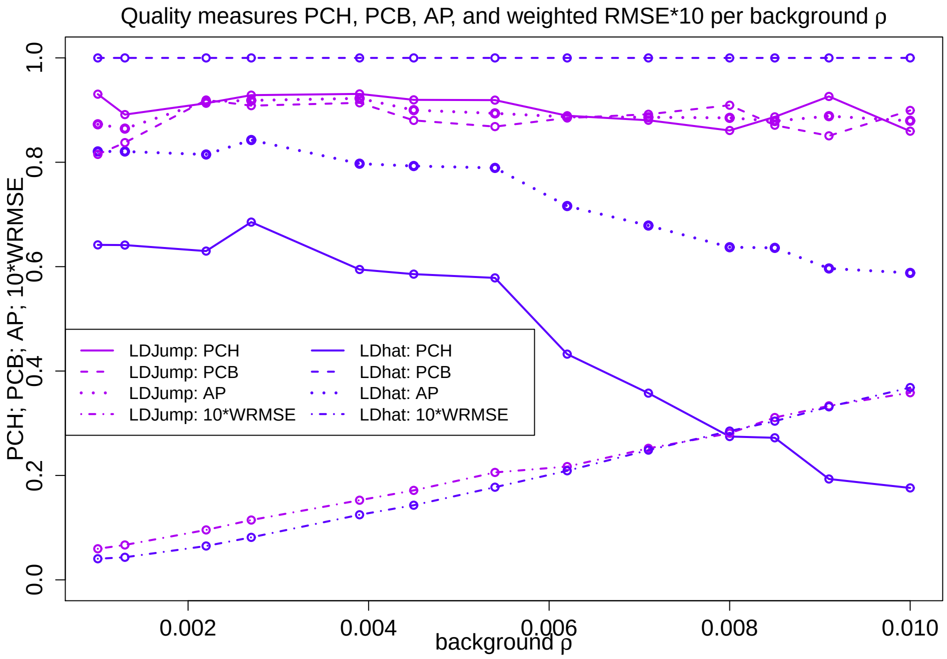

Figure 10: Proportion of correctly identified hotspots (PCH, solid), proportion of correctly identified background rates (PCB, dashed), the average of these two quality measures (AP, dotted), and weighted RMSE*10 (dash-dotted) across different recombination rates. We compare *LDJump* (purple, segment length: 1kb, quantile 0.35), with *LDhat* (blue, same line coding per quality measure).

4 Quality Assessment for Natural Setups with *FastEPRR*

Here we compare the results of *LDJump* with *FastEPRR* based on the *natural* setups. Notice that due to the very high error share of 88% in *FastEPRR* using segment lengths of 1kb we only compare the results of actually estimated recombination maps. For the sake of visibility, we assess *LDJump* using our recommended quantile of 0.35 in the bias correction and compare across the number of segments of 500, 1000, 1500, and 2000. Figure 11 shows that *LDJump* estimates recombination maps with smaller WRMSE, irrespective of the segment lengths considered and has a much higher share of correctly identified hotspots (PCH), but a lower share of correctly identified background rates (PCB).

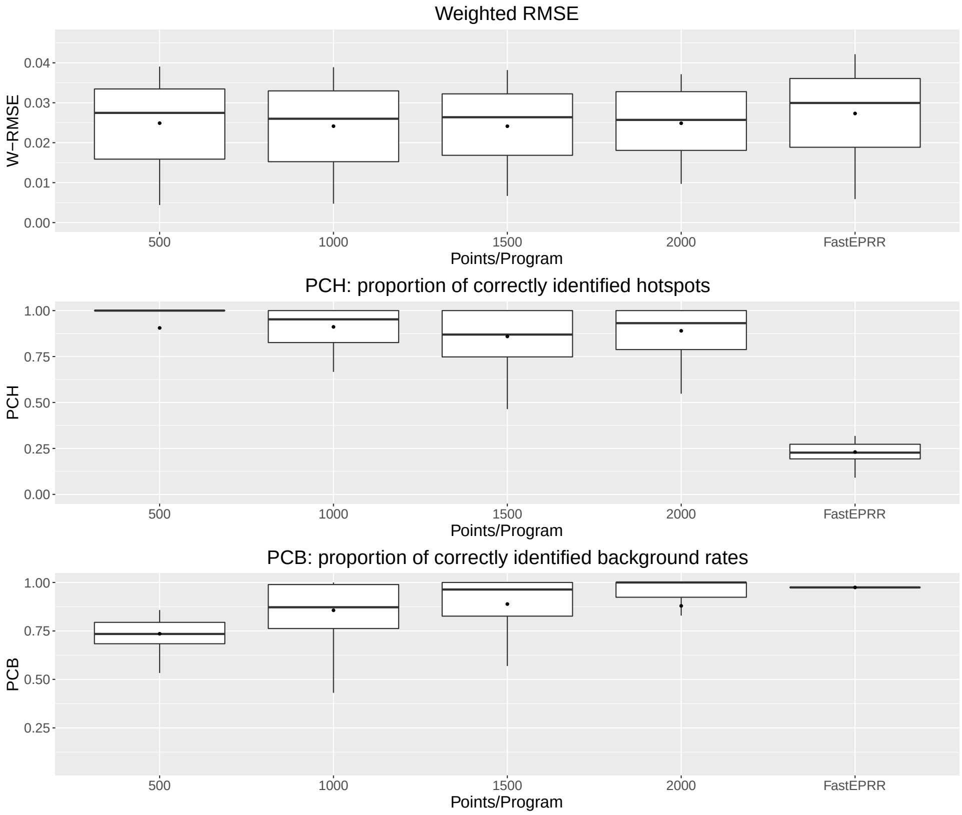

Figure 11: Natural setups: quality assessment is performed based on the weighted RMSE (top), the proportion of correctly identified hotspots (PCH, middle), and the proportion of correctly identified background rates (PCB,bottom). The results for *LDJump* were computed using different number of initial segments k (500, 1000, 1500, 2000) and compared with the results of *FastEPRR* using segment lengths of 1000 base pairs.

5 Runtime Comparison

5.1 Runtime under Simple Setups

Based on the summary statistics mean (top), median (middle), and SD (bottom) of our measured runtimes we compare the runtimes between the considered software packages in Table 3. We can clearly see that *LDJump* has the smallest runtime followed by *FastEPRR, LDhat(v1), LDhat*, and *LDhelmet*.

	LDhat(v1) (*bpen*)			*LDhat*(*bpen*)			*LDhelmet*(*bpen*)			*FastEPRR* (seg. length)				*LDJump* (*k*)			
	0	5	50	0	5	50	0	5	50	500	1000	1500	2000	10	20	25	30
$\bar{x}$	35	56	156	751	3333	3260	1281	1368	1958	139	113	85	65	27	39	45	49
$x_{0.5}$	34	55	138	735	3315	3261	849	936	1575	121	131	98	77	28	42	48	53
SD	6	7	70	273	999	977	1034	1042	1125	84	40	28	21	5	8	9	10

Table 3: Mean ($\bar{x}$), median ($x_{0.5}$), and SD of runtime (in seconds) for *LDhat(v1)*, *LDhat* , *LDhelmet*, *FastEPRR*, and *LDJump* under simple setups of length 20kb. For each method, separate columns provide values depending on either the block penalty for *LDhat(v1), LDhat, LDhelmet* (columns 2-4, 5-7, 8-10, respectively), the segment length (seg. length) for *FastEPRR* (columns 11-14) or the number of predefined segments k on which *LDJump* was applied (columns 15-18).

5.2 Effect on Runtime by Increasing Sample Size and Sequence Length

In Table 4 we explore the effects of sample size and sequence length on the runtime. We compared the aforementioned methods with respect to their mean and median runtimes again for our *simple* setups. The runtimes for *LDhat* and *LDhelmet* are strongly affected by sequence length and sample size. Interestingly *LDhat* seems to have more problems dealing with longer sequences, whereas *LDhelmet* shows an especially large increase in runtime when the sample size increases. The runtime of *LDJump* (using segments of length 500 and 1000 bp) seems to be less sensitive to such increases. Doubling the sequence length only leads to additional 16% of average runtime. Increasing the sample size has almost no effect on the runtime of *LDJump*. We observe a similar behavior of *FastEPRR* (using a segment length of 1kb) with more pronounced effects on the double "initial" runtime for the smallest sample size and sequence lengths.

5.3 Runtime under Natural Setups

Table 5 shows average and median runtimes in seconds per 20 replicates of the 13 different *natural* setups. In the first five rows we provide the mean runtimes of *LDJump* with k = 500, 1000, 1500, and 2000, and of *LDhat*. The same pattern builds rows 6-10 for the median. The columns show the increasing background rates and highlight that the mean and (to a larger extent) the median of *LDhat* is more strongly affected by larger recombination rates than *LDJump* with approximately constant runtimes across these setups. The runtime of *LDJump* is mainly determined by the computation of the summary statistics. However, *LDJump* is

Time	Method	Sample Size			Sequence Length	
		10	16/10	20/10	10kb	20kb/10kb
Mean	*LDhat(v1)*	124	141	149	121	155
Mean	*LDhat*	1862	2484	2634	1388	3262
Mean	*LDhelmet*	709	1347	3247	1581	1960
Mean	*FastEPRR*	88	88	90	76	113
Mean	*LDJump*	37	36	37	34	34
Median	*LDhat(v1)*	109	125	135	111	138
Median	*LDhat*	1526	1969	2075	1398	3257
Median	*LDhelmet*	625	1219	3225	1101	1574
Median	*FastEPRR*	74	80	83	78	131
Median	*LDJump*	35	35	36	34	42

Table 4: Runtime in seconds are provided separately for different sample sizes and sequence lengths. We computed the mean and median runtime for each method and scenario under *simple* setups.

approximately (depending on the number of segments chosen) between 340 and 1400 times faster than *LDhat*.

Runtime	Method	Background rates per base pair												
		0.001	0.0013	0.0022	0.0027	0.0039	0.0045	0.0054	0.0062	0.0071	0.008	0.0085	0.0091	0.01
Mean	*LDJump*, k=500	54	55	54	55	55	55	55	55	55	55	54	54	56
Mean	*LDJump*, k=1000	111	111	110	111	110	111	112	111	111	111	111	112	112
Mean	*LDJump*, k=1500	169	169	166	167	167	167	168	168	167	168	167	167	168
Mean	*LDJump*, k=2000	226	225	222	223	224	225	229	225	222	224	226	224	223
Mean	*LDhat*	73902	73752	77171	87025	73832	74423	80707	70203	86679	81078	70239	74016	81053
Median	*LDJump*, k=500	54	55	54	55	55	55	55	55	55	55	54	54	56
Median	*LDJump*, k=1000	111	111	110	111	110	111	112	111	111	111	111	112	112
Median	*LDJump*, k=1500	169	169	166	167	167	167	168	168	167	168	167	167	168
Median	*LDJump*, k=2000	226	225	222	223	224	225	229	225	222	224	226	224	223
Median	*LDhat*	100963	100963	124040	125921	100963	100934	125856	98629	126433	126072	98629	100963	126072

Table 5: Mean and median of runtime (in seconds) are provided for each approach. The runtimes in seconds applying *LDJump* with $k = 1000, 1500, 2000$, and using *LDhat* are compared across all considered background recombination rates.

6 Application of *LDJump* on chromosome 21 under neutrality

Figure 12 shows the application of *LDJump* with segment lengths of 1kb, a quantile of 0.35, and under the neutral scenario (estimated without considering demography). *LDJump* estimates hotspots of high intensities for several positions not overlapping with *LDhat* or active recombination measures. In comparison, several of these hotspots (e.g. at positions 45 and 60

kb for TSI or 25 and 30 for FIN, respectively) were not estimated by *LDJump* trained using demography model (2) (see panel A in Figure 6 of the main manuscript). Hence, including demography in the estimation of hotspots from LD is an important feature to reduce false positives.

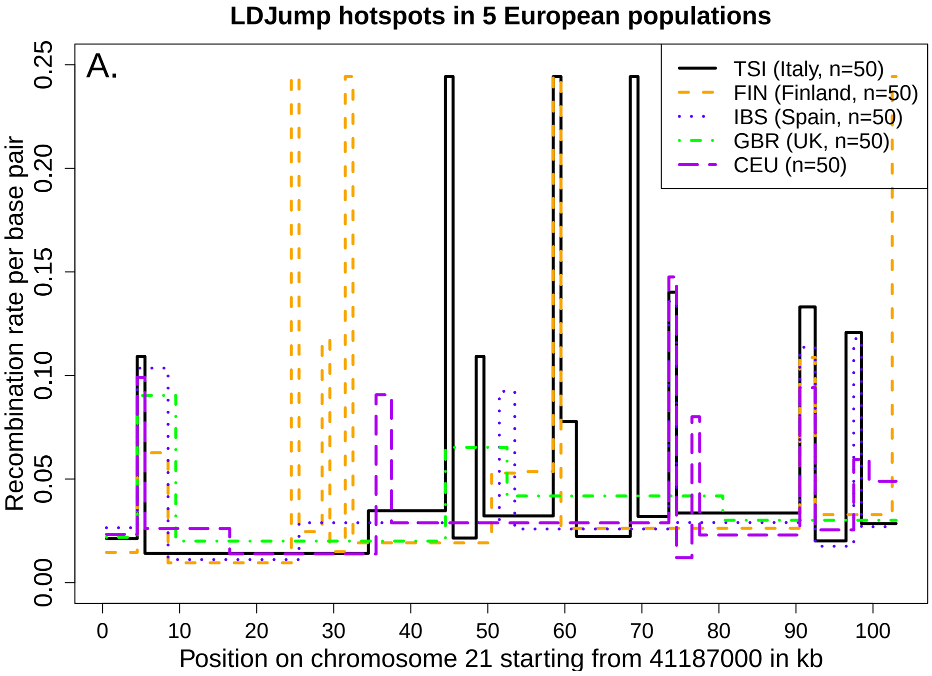

Figure 12: Estimated recombination map with *LDJump* (under neutrality) of 5 different European populations (Italy, Finland, Spain, United Kingdom, Northern Europeans from Utah - CEU) on chromosome 21:41187000-41290679 (GRCH37).

References

[Birdsell, 2002] Birdsell, J. A. (2002). Integrating Genomics, Bioinformatics, and Classical Genetics to Study the Effects of Recombination on Genome Evolution. Molecular Biology and Evolution, 19(7):1181–1197.

[Box and Cox, 1964] Box, G. E. P. and Cox, D. R. (1964). An analysis of transformations. Journal of the Royal Statistical Society. Series B (Methodological, pages 211–252.

[Duret and Galtier, 2009] Duret, L. and Galtier, N. (2009). Biased Gene Conversion and the Evolution of Mammalian Genomic Landscapes. Annual Review of Genomics and Human Genetics, 10(1):285–311.

[Frick et al., 2014] Frick, K., Munk, A., and Sieling, H. (2014). Multiscale change-point inference. Journal of the Royal Statistical Society: Series B, 76(3):495–580.

[Jombart, 2008] Jombart, T. (2008). adegenet: a R package for the multivariate analysis of genetic markers. Bioinformatics, 24(11):1403–1405.

[Pages et al., 2016] Pages, H., Aboyoun, P., Gentleman, R., and DebRoy, S. (2016). Biostrings: String objects representing biological sequences, and matching algorithms.

[Paradis et al., 2004] Paradis, E., Claude, J., and Strimmer, K. (2004). A{PE}: analyses of phylogenetics and evolution in {R} language. Bioinformatics, 20:289–290.

[R Development Core Team, 2017] R Development Core Team (2017). R: A language and environment for statistical computing.
